# Colon Capsule Endoscopy in Detecting Colonic Diverticula in a Japanese Population

**DOI:** 10.7759/cureus.12393

**Published:** 2020-12-31

**Authors:** Konosuke Nakaji

**Affiliations:** 1 Internal Medicine: Gastroenterology, Endoscopy Center, Aishinkai Nakae Hospital, Wakayama-Shi, JPN

**Keywords:** colon capsule endoscopy, colonic diverticula, epidemiology

## Abstract

Objective

The assessment of colonic diverticula with colon capsule endoscopy (CCE) in a Japanese population provided unclear results. In this study, we retrospectively reviewed a cohort of Japanese patients who had undergone CCE to assess its safety and usefulness in the diagnosis of colonic diverticula.

Methods

In this study, 175 consecutive Japanese patients who had their entire colon observed via CCE from November 2013 to July 2018 were included. Patients were retrospectively stratified according to age, gender, colonic segment, and symptoms involvement. A multivariable regression analysis was performed to investigate the presence of any correlation among variables. The safety of CCE was assessed in terms of the incidence of adverse events (AEs).

Results

Colonic diverticula were observed in 42.3% of all cases; of those; 36.5% were right-sided, 31.1% were left-sided, and 32.4% were bilateral. Moreover, one to two colonic diverticula were observed in 35.1%, while three or more diverticula were seen in 64.9%. Multivariable analysis showed that age (≥70 years) was positively associated with colonic diverticula, while male gender and the presence of colonic polyps were negatively associated with colonic diverticula. No correlation was found between colonic diverticula and symptoms. There was no significant difference between groups with and without colonic diverticula in the incidence of AEs. AEs were mild in severity, with no severe AE-related bowel preparation and capsule ingestion reported.

Conclusion

CCE was well-tolerated by the participants, and the incidence of colonic diverticula was 42.3%, with one to two and three or more diverticula being found in 35.1% and 64.9%, respectively. There was little difference in the frequency of colonic diverticula formation on the right side, left side, and on both sides. Age was a positive association factor, while male gender and the presence of colorectal polyps were negative association factors. No correlation was found between diverticula and symptoms.

## Introduction

In recent years, the prevalence of colonic diverticula has increased in Japan [1]. As a consequence of this increase, the incidence of emergent management of diverticular diseases of the colon, such as diverticular bleeding [2] and diverticulitis [3] of the colon, will rise in the setting of daily medical care. Therefore, the appropriate and timely diagnosis of colonic diverticula is critical. Recent literature has evaluated the epidemiology of colonic diverticula with barium enema [4], colonoscopy (CS) [4], and CT colonography (CTC) [5]. However, to the best of our knowledge, there are no reports of an epidemiological study of colonic diverticula assessed with colon capsule endoscopy (CCE). The use of second-generation CCE has become clinically viable in recent years [6], and it is a non-invasive and simple examination that only requires the patient to swallow a capsule [7]. In Japan, CCE is mainly used for the detection of colonic polyps [8] and the evaluation of mucosal lesions in patients with inflammatory bowel disease [9] where CS is difficult to perform [7]. Since the colonic mucosa can be observed spontaneously without insufflation, we have found that it is clearly visible even in small colonic diverticula visualized using CCE. Therefore, we believe CCE is feasible for detecting colonic diverticula. Hence, we retrospectively evaluated a cohort of Japanese patients who had their colonic diverticula visualized with CCE to examine the usefulness of CCE for detecting colonic diverticula.

## Materials and methods

Patient selection

In this retrospective study, we included consecutive Japanese patients who had their entire colon observed via CCE (having confirmation of the colon capsule discharged within the examination time or observation of the dentate line) from November 2013 to July 2018 at our institute. These patients underwent CCE due to difficulties in undergoing total CS (observation of anus to cecum by CS), which were defined as anticipation of difficulty in CS due to intestinal adhesion or incompletion of total CS. Patients who had dysphasia, those who had an inserted cardiac pacemaker, or those who were pregnant were excluded from this study. The study was conducted in accordance with the 2013 Declaration of Helsinki and was approved by the Ethics Committee of Aishinkai Nakae Hospital, Wakayama, Japan (#017). Written informed consent was obtained from all patients.

Evaluation of risk factors and adverse events

We evaluated the prevalence of colonic diverticula by investigating their location, number, and possible risk factors associated with the formation of colonic diverticula including age, sex, the colon transit time of capsule, abdominal pain, hematochezia, abnormal bowel habits (suffering from constipation that required prescription of laxatives and/or suffering from diarrhea), fecal occult blood tests (FOBT), the presence of colonic polyps, and adverse events (AEs) related to bowel preparation and capsule ingestion.

CCE procedure

**Figure 1:**
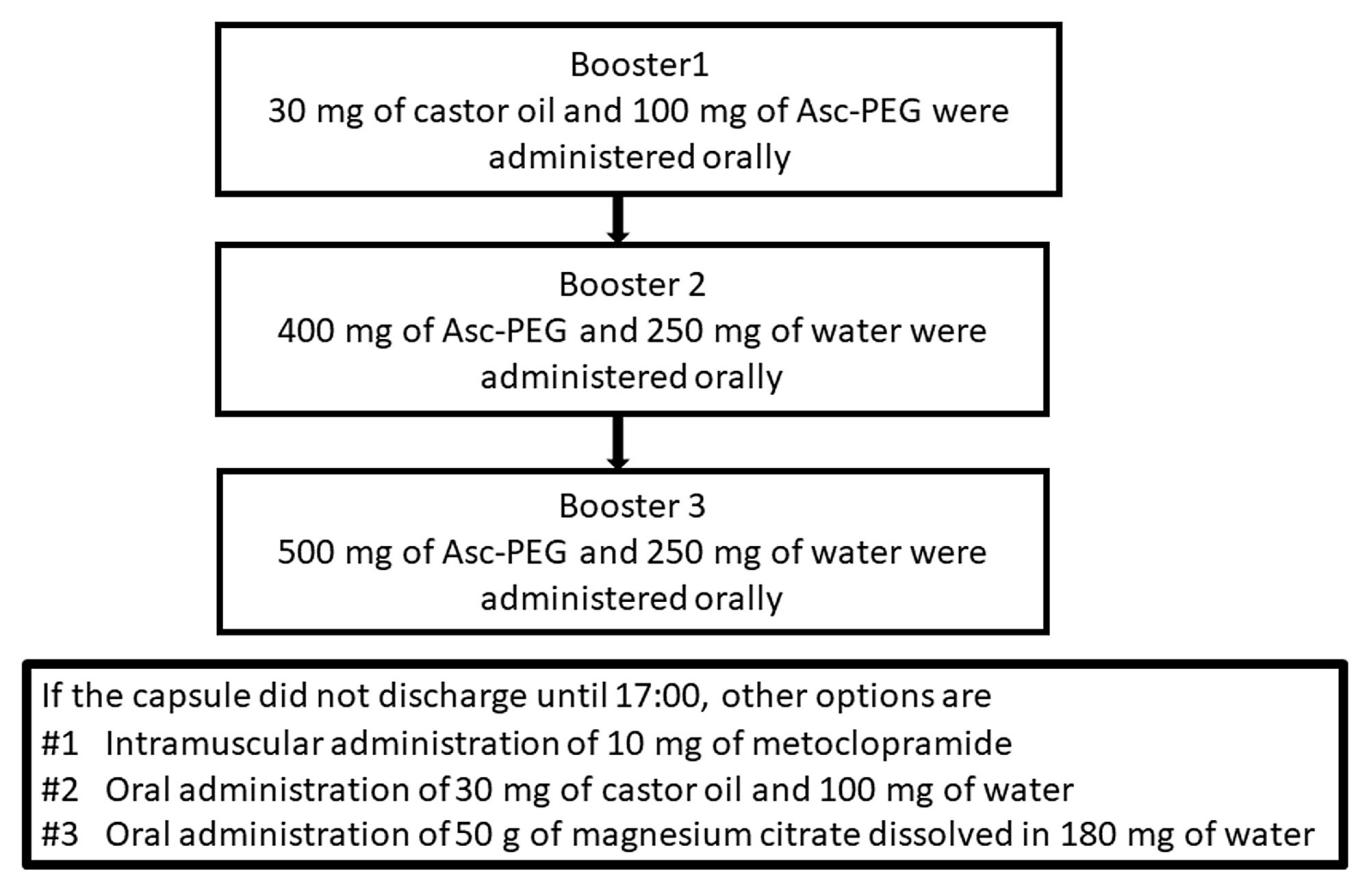
Boosters for the discharge of colon capsule Asc-PEG: ascorbic acid-containing polyethylene glycol solution

The colon capsule was treated with PillCamCOLON2 (Medtronic, Minneapolis, MN) in all cases. The pretreatment of CCE at our institute was performed as follows: if constipated, patients were required to take 36 milligrams (mg) of sennoside at bedtime two days before the examination (in case of chronic constipation, patients were required to take 36 mg of sennoside every day for one week prior to the examination). The day before the examination, patients ingested a low-residue diet at home, and in the evening, they took a hypertonic solution prepared by dissolving 50 g of magnesium citrate (Magcorol P; Horii Pharmaceutical Co., Ltd., Osaka, Japan) in 180 mg of water. At bedtime, 10 mg of 0.75% sodium picosulfate and 80 mg of water were taken by the patients. On the day of the examination, patients took 500-1,000 mg of ascorbic acid-containing polyethylene glycol solution (Asc-PEG; EA Pharma Co., Ltd, Tokyo, Japan) and 250-500 mg of water one to two hours before oral administration of the capsule until the stool became clear and liquid, and they swallowed a colon capsule after the administration of 20 mg of mosapride. During the examination, patients were asked to walk to promote intestinal peristalsis or to maintain the right lateral decubitus position. If the capsules were retained in the stomach for one hour according to a real-time viewer, 10 mg of metoclopramide was administered intramuscularly to facilitate gastric emptying. Additional treatment with laxatives, e.g., boosters, was administered in order to promote the discharge of capsules. When the capsule reached the duodenum, 30 mg of castor oil and 100 mg of Asc-PEG were administered orally as booster 1. Later, if the colon capsule was not discharged, 400 mg of Asc-PEG and 250 mg of water were administered orally as booster 2, and 500 mg of Asc-PEG and 250 mg of water were administered orally as booster 3. If the capsule did not discharge until 17:00, other options included intramuscular administration of 10 mg of metoclopramide, oral administration of 30 mg of castor oil and 100 mg of water, or an oral administration of 50 g of magnesium citrate dissolved in 180 mg of water (Figure 1). Finally, 60 mg of glycerin enema was administered if there was no discharge of the colon capsule. These steps are based on the recommended pretreatment regimen of CCE for colonic polyp detection by the Japanese Association for Capsule Endoscopy (JACE).

Review of CCE findings for colonic diverticula

After the examination was completed, the data recorder (DR3) was downloaded to workstations that included a dedicated interpretation software (RAPID software v8.0 or v8.3). Colon capsule endoscopic findings were reviewed by a JACE-certified support technician and an experienced physician (JACE supervising physician) (NK). The evaluation of colonic diverticula was performed not only by single capsule endoscopy findings from captured images in the thumbnail section but also by reviewing the entire data again and trying not to overlook the colonic diverticula.

Statistical analysis

Mean ± standard deviation or percentage was used for all data. In univariate analysis. Pearson’s chi-squared test was used when investigating the relationship between the presence of diverticula and the variables of mean age and colon transit time. Fisher’s exact test was used when investigating the relationship between the presence of diverticula and the variables of the female gender, FOBT positivity, abdominal pain, hematochezia, abnormal bowel habits and weight loss, colonic polyp, and AEs.

Multivariable analysis was performed using multiple regression analysis using the presence or absence of colonic diverticula as an independent variable. A p-value of <0.05 was considered statistically significant for all tests. Statistical analysis was performed using IBM SPSS Statistics software (IBM, Armonk, NY).

## Results

Demographics of the patients

During the study period, 206 consecutive Japanese patients underwent CCE, of which 175 patients who had their entire colon successfully observed were available for analysis. The mean age of the patients was 62.0 ± 14.9 years (range: 19-90 years), with 91 men and 84 women. The most common reason for CCE was screening (n=59). Indications for CCE included 159 cases of anticipation of difficulty in performing CS and 16 cases of incomplete CS (Table 1).

Prevalence and numbers of colonic diverticula as assessed by CCE

**Figure 2:**
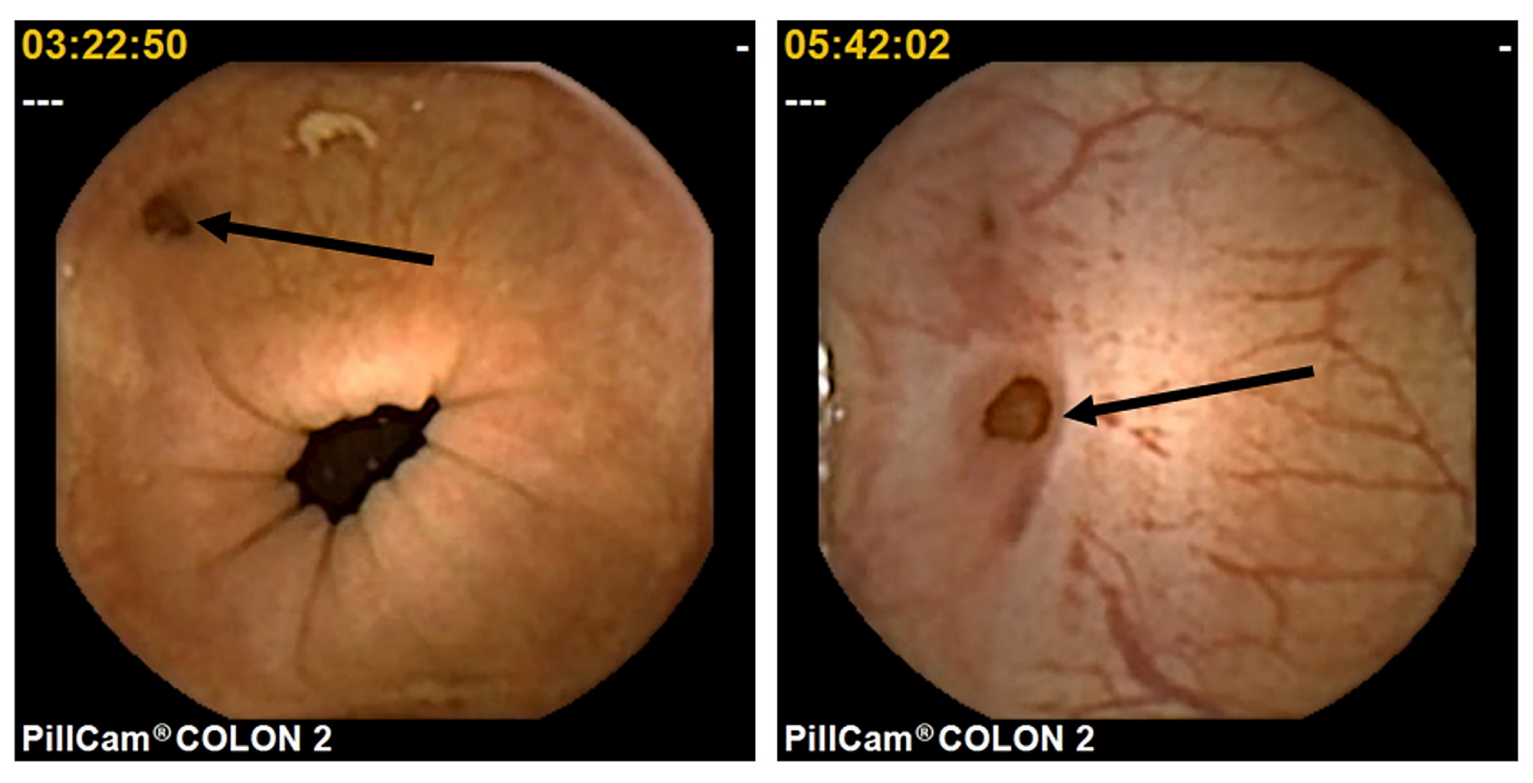
Representative image of colonic diverticula observed via CCE (arrows) CCE: colon capsule endoscopy

**Figure 3:**
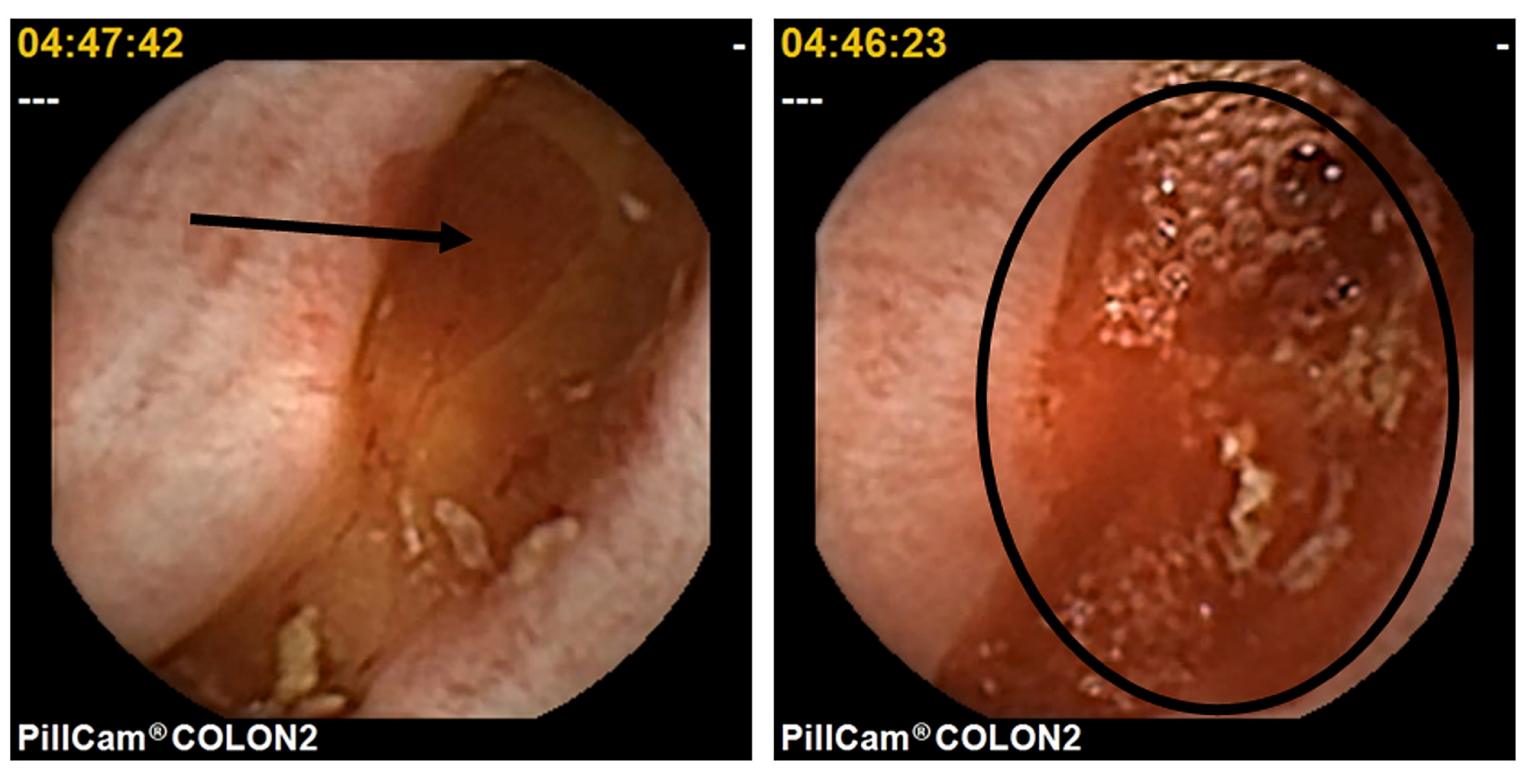
Representative image of colonic diverticular bleeding observed via CCE (arrow and circle) CCE: colon capsule endoscopy

The frequency of colonic diverticula was 42.3% (74/175 patients). Moreover, one to two colonic diverticula and three or more colonic diverticula were found in 35.1% and 64.9% of patients, respectively. Representative CCE images of colonic diverticula and a case of colonic diverticular bleeding from this study are shown in Figure 2 and Figure 3, respectively.

Table 1 shows the patient characteristics of groups with or without colonic diverticula. In the univariate analysis of colonic diverticulum and each factor, the age of the patients was significantly higher in the group with colonic diverticula (p=0.010). The frequency of colonic diverticula was also significantly higher in females (p=0.014). There was no significant association between the colonic transit time of the capsule, abdominal pain, hematochezia, abnormal bowel movement, or positive or negative FOBT results, and the presence and absence of colonic diverticula. Colonic polyps were significantly more common in the group without diverticulosis (p=0.021).

**Table 1:** Characteristics of the patients and results of univariate analysis assessing the factors associated with colonic diverticula SD: standard deviation; FOBT: fecal occult blood test

Variables	Overall cases (n=175)	With diverticula (n=74)	Without diverticula (n=101)	P-value
Age (years), mean ± SD	62.0 ± 14.9	65.3 ± 15.0	59.5 ± 14.4	0.010
Female gender, n (%)	84 (48.0%)	44 (59.5%)	40 (39.6%)	0.014
Symptoms				
FOBT-positive, n (%)	30 (17.1%)	13 (17.6%)	17 (16.8%)	1.000
Abdominal pain, n (%)	25 (14.3%)	15 (20.3%)	10 (9.9%)	0.079
Hematochezia, n (%)	6 (3.4%)	1 (1.4%)	5 (49.5%)	0.403
Abnormal bowel habits, weight loss, n (%)	12 (6.9%)	4 (5.4%)	8 (7.9%)	0.563
Indications				
Anticipated difficulty of total colonoscopy, n (%)	159 (90.9%)	64 (86.5%)	95 (94.1%)	
Failure of total colonoscopy, n (%)	16 (9.1%)	10 (13.5%)	6 (59.4%)	
Colon transit time (minutes), mean ± SD	170.4 ± 103.0	178.3 ± 94.7	164.7 ± 108.9	0.38
Sites of colonic diverticula				
Right-sided only, n (%)		14 (18.9%)		
Left-sided only, n (%)		42 (56.8%)		
Both sides, n (%)		24 (32.4%)		
Number of diverticula				
One to two, n (%)		26 (35.1%)		
Over three, n (%)		48 (64.9%)		
Colonic polyp, n (%)	80 (45.7%)	26 (35.1%)	54 (53.5%)	0.021

Locations of colonic diverticula and other findings

Locations of colonic diverticula in our study were as follows: cecum (14 cases), ascending colon (42 cases), transverse colon (two cases), and sigmoid colon and descending colon (48 cases); none was found in the rectum. Regarding the different types of colonic diverticula, 27 patients (36.5%) had the right-sided type (proximal to the splenic flexure), 23 patients (31.1%) had the left-sided type (distal to the splenic flexure), and 24 patients (32.4%) had the bilateral type. Apart from the colonic diverticula, there were additional findings such as colonic polyps (n=80), colonic cancer (n=5), and colonic angioectasia (n=14).

Relationship between colonic diverticula and risk factors

**Figure 4:**
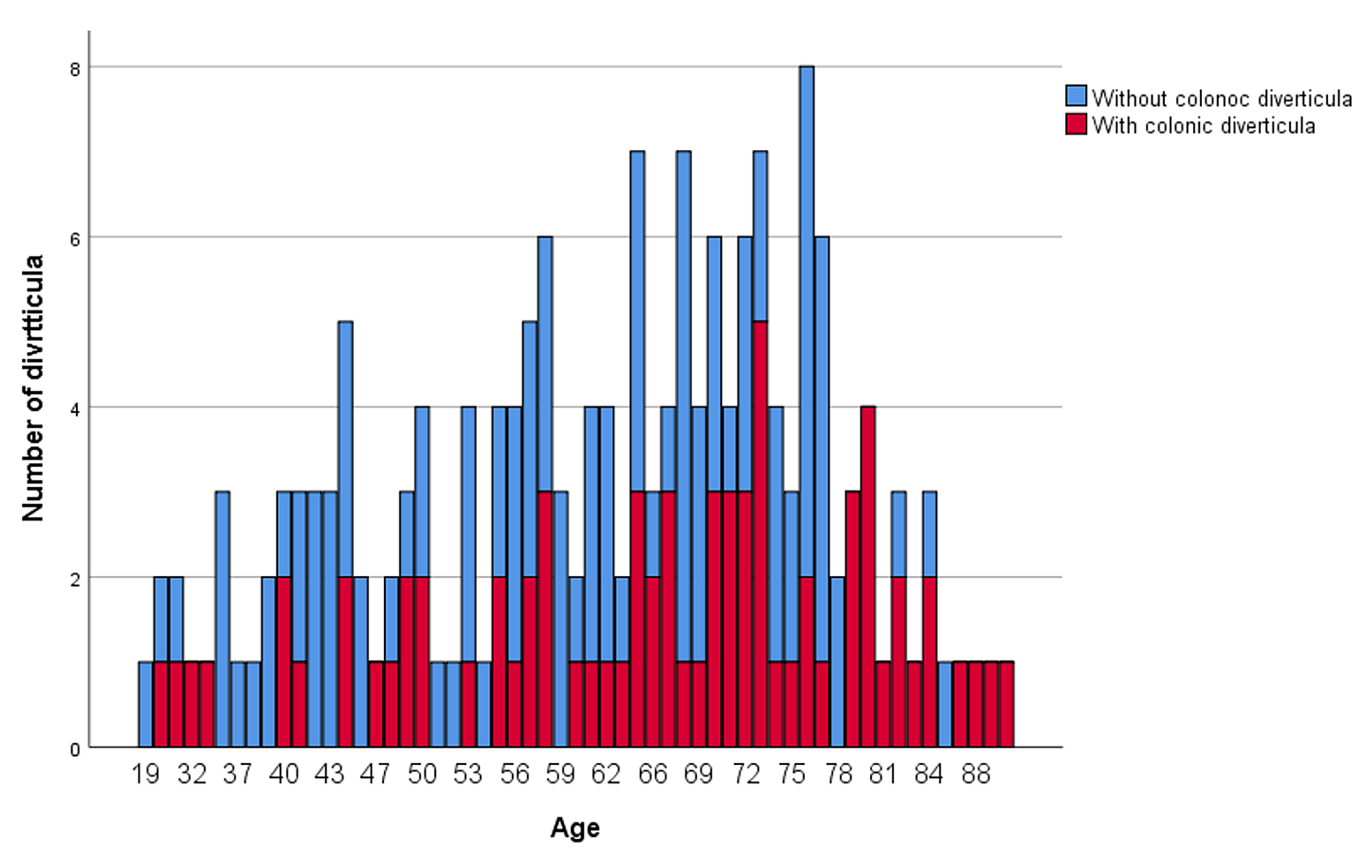
Prevalence of colonic diverticula stratified by age group

**Figure 5:**
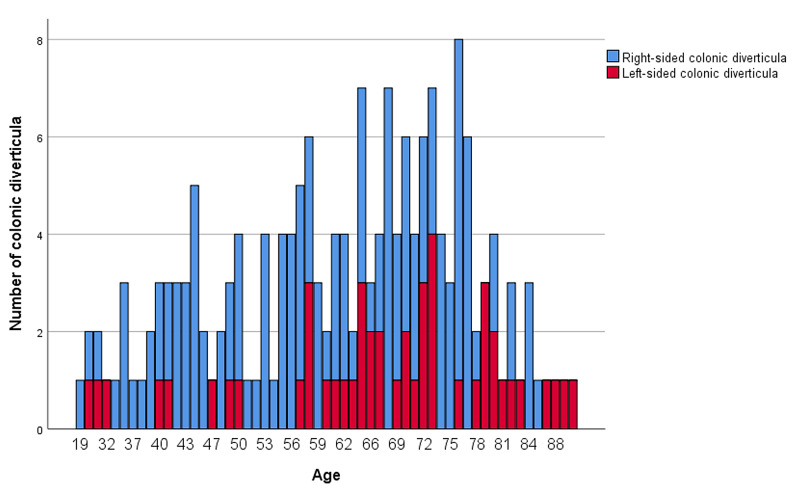
Proportion of right- and left-sided colonic diverticula stratified by age group

The prevalence of colonic diverticula was found to increase with age (Figure 4), and the prevalence of left-sided diverticula, specifically, also increased with age (Figure 5).

In the multivariable analysis (multiple regression analysis), the presence or absence of colonic diverticula was used as an independent variable, while age (≥70 years), colorectal polyps, male sex, and abdominal pain were set as dependent variables. It was found that age (≥70 years) influenced the propensity of colonic diverticula to develop (B=0.262, t-value=3.505, p=0.001), and colonic polyps and male gender affected the propensity of colonic diverticula to lack colorectal diverticula (colorectal polyps: B=−0.915, t-value=−2.624, p=0.009; male sex: B=−0.162, t-value=2.243, p=0.026) (Table 2).

**Table 2:** Multivariable analysis of the risk factors associated with colonic diverticula B: partial regression coefficient; SE: standard error

Risk factors	B	SE	t-value	P-value
Age (≥70 years)	0.262	0.075	3.505	0.001
Colonic polyp (positive)	-0.195	0.074	-2.624	0.009
Gender (male)	-0.162	0.072	-2.243	0.026
Abdominal pain	0.14	0.102	1.365	0.174

The detection rate of CCE

In comparing the observed regions in CCE and the previous 62 colonoscopies, the detection rate of CCE for colonic diverticula was 100% with at least one diverticulum matched.

Safety

Table 3 shows the incidence rate of AEs; no significant difference was observed between the group with colonic diverticula and that without (1.35% vs. 0.99%) (p=1.00). AEs were of mild severity, with no severe AE such as intestinal obstruction due to capsule retention or examination discontinuation due to AE reported. No death occurred during the study period. One patient in the group without colonic diverticula experienced nausea and vomiting during the procedure. One patient in the colonic diverticula group could not swallow the colon capsule. Subsequently, the colon capsule was led to the duodenum endoscopically with the retrieval nets, and CCE was completed successfully.

**Table 3:** List of adverse events (safety analysis) AE: adverse event; SAE: serious adverse event

Adverse events	With diverticula (n=74)	Without diverticula (n=101)
Any AE	1 (1.35%)	1 (0.99%)
Any SAE	0	0
AEs related to bowel preparation		
Nausea, vomiting	0	1
AEs related to capsule ingestion	1	0

## Discussion

In this study, the prevalence of colonic diverticula in a Japanese population was found via CCE to be 42.3%. While age was a positive association factor, male gender and the presence of colorectal polyps were negative association factors. To the best of our knowledge, this is the first study to investigate the prevalence and risk factors influencing the etiology of colorectal diverticula using CCE in a Japanese population. The prevalence of colonic diverticula in the Japanese population has been considered to be generally lower than that in the Western population. In 2011, the prevalence of colonic diverticula in Japanese general hospitals was reported to be 23.0%. In contrast, the prevalence of colonic diverticula in the United States in patients with an average age of 55 years was 42-60% [1]. In the present study, the prevalence of colonic diverticula in a Japanese population was similar to that in patients in Western countries. Although making a generalization is difficult because of the small sample size and the retrospective, single-institution design of this study, the current prevalence of colonic diverticula in Japan may indeed be similar to that in Europe and the United States.

In a previous study of 4,386 individuals in northern China in 2018 regarding gender differences in colonic diverticula, Yang et al. reported a high incidence in the number of colonic diverticula in males who underwent CS evaluation (p=0.013) [10]. Yamaguchi et al. reported a high incidence of colonic diverticula in males in 2015 in a CS epidemiological analysis of 62,503 Japanese patients (p=0.0011) [11]. In contrast, in a recent paper on Western populations, it has been reported that in young populations, colonic diverticula are more common in males, but in older populations, they are more common in females [12]. In contrast with previous reports, this study found more cases of colonic diverticula in females than in males. In Japan, colonic diverticula might become more common in females going forward, due to the westernization of their lifestyle and the aging of the population. Moreover, since a study [13] reported that females tend to expel the colon capsule more slowly, it is possible that there was a bias since the capsule remaining in the colon for a longer time period can cause colonic diverticula to be more easily discovered.

In this study, 36.5% of the diverticula were found on the right side, 31.1% were on the left side, and 32.4% were of the bilateral type. In a study of diverticula sites in 2015, Wang et al. reported that the incidence of diverticula was 52.3% on the right side, 25% on the left side, and 22.7% on both sides in 1,889 Taiwanese patients [14]. The results of this study showed little difference in the frequency of colonic diverticula formation on the right side, left side, and both sides. The reason for this is unknown, but we speculate that the modality of capsule endoscopy being involved in shunting the right- and left-sided colon through the splenic flexure cannot be ruled out.

Regarding the relationship between age and the location of diverticula, the proportion of patients with diverticula has tended to be higher in the Japanese cohort with younger age as a risk factor for colonic diverticula on the right side and older age as a risk factor for colonic diverticula on the left side. The same trend was observed in this study. On the other hand, in Europe and the United States, 80% of colonic diverticula were found to be present on the left side of the colon, and the sigmoid colon was the most common site, accounting for 70% of the diverticula. The frequency increases with age, but it has been reported that the proportion of diverticula in the left and right colon does not change significantly [1]. This may be due to differences in anatomical findings between races and the involvement of neurohormonal systems.

In an analysis of the number of colonic diverticula in patients with a mean age of 55 years in the United States, three or more diverticula were found in 28% of patients, one or two diverticula were found in 32%, and the absence of diverticula was reported in 40% [1]. In this study, one to two diverticula were found in 35.1% of patients, while three or more diverticula were found in 64.9%. It is noteworthy that the age of the patients in this study was higher than that reported in studies in Europe and the United States.

Regarding the relationship between colonic diverticula and symptoms, Yamada et al. have reported that constipation and colonic diverticula were not significantly associated, but constipation was negatively associated with the presence of a left colonic diverticulum [15]. In the present study, no relationship was found between colonic diverticula and symptoms such as abdominal pain, hematochezia, and abnormal bowel habits. There have been reports of a positive association between colonic polyp/cancer and diverticula in the past [16-19], but recently, a study has reported no relationship between colonic diverticula and colorectal polyp/cancer [20]. In addition, colonic diverticula were more common in the group without colonic polyps in this study. Further studies should address this issue in the future.

In comparing the images between CCE and other modalities, as reported recently in a study in Japan [21], the detection rate of colonic diverticula of barium enema, CS, and CTC was 37.5%, 23.9%, and 56.8%, respectively. This suggested that CCE might be a sensitive method of detecting colonic diverticula, but inferior to CTC. However, CCE is free from radiation exposure and there is no risk of perforation as compared to CTC.

Compared to the group without colonic diverticula, the group with the presence of colonic diverticula demonstrated a favorable safety profile and a higher level of toleration, which no significant difference in the incidence of AEs between the groups. Most AEs were mild in severity, and no death was reported. The known complications related to bowel preparation and capsule ingestion were reported in only a few cases in this study. The incident rate of minor complications for CCE in the presence of colonic diverticula (1.35%) was lower than previously reported in the general population (4.1%) [7].

This study has several limitations. Firstly, this was a small retrospective study. Secondly, CCE often depicts an intermittent depressed image around a penetrating vessel, the so-called pseudo diverticulum. Thirdly, the detection rate of CCE for colonic diverticula may depend on capsule transit time and location. Finally, the same diverticula were detected multiple times, and therefore the differentiation of unique diverticula may be difficult.

## Conclusions

Our study showed that CCE was well-tolerated by patients, and the prevalence of colonic diverticula in a Japanese population was 42.3%, with the incidence of right-sided, left-sided, and bilateral types being 36.5%, 31.1%, and 32.4%, respectively. One to two and three or more diverticula were found in 35.1% and 64.9% of the patients, respectively. Age (≥70 years) was positively associated with colonic diverticula, while male gender and the presence of colonic polyps were negatively associated. No relationship was found between colonic diverticula and symptoms.
